# Autonomous Robot for Removing Superficial Traumatic Blood

**DOI:** 10.1109/JTEHM.2021.3056618

**Published:** 2021-02-02

**Authors:** Baiquan Su, Shi Yu, Xintong Li, Yi Gong, Han Li, Zifeng Ren, Yijing Xia, He Wang, Yucheng Zhang, Wei Yao, Junchen Wang, Jie Tang

**Affiliations:** 1 Medical Robotics Laboratory, School of AutomationBeijing University of Posts and Telecommunications 12472 Beijing100876China; 2 Department of GastroenterologyPeking University Third Hospital 66482 Beijing100191China; 3 School of Mechanical Engineering and AutomationBeihang University 12633 Beijing100191China; 4 Beijing Advanced Innovation Center, Biomedical EngineeringBeihang University 12633 Beijing100086China; 5 Department of NeurosurgeryXuanwu HospitalCapital Medical University 12517 Beijing100053China

**Keywords:** Blood removal, contour detection, medical robot, task autonomy

## Abstract

*Objective*: To remove blood from an incision and find the incision spot is a key task during surgery, or else over discharge of blood will endanger a patient’s life. However, the repetitive manual blood removal involves plenty of workload contributing fatigue of surgeons. Thus, it is valuable to design a robotic system which can automatically remove blood on the incision surface. *Methods*: In this paper, we design a robotic system to fulfill the surgical task of the blood removal. The system consists of a pair of dual cameras, a 6-DoF robotic arm, an aspirator whose handle is fixed to a robotic arm, and a pump connected to the aspirator. Further, a path-planning algorithm is designed to generate a path, which the aspirator tip should follow to remove blood. *Results*: In a group of simulating bleeding experiments on ex vivo porcine tissue, the contour of the blood region is detected, and the reconstructed spatial coordinates of the detected blood contour is obtained afterward. The BRR robot cleans thoroughly the blood running out the incision. *Conclusions*: This study contributes the first result on designing an autonomous blood removal medical robot. The skill of the surgical blood removal operation, which is manually operated by surgeons nowadays, is alternatively grasped by the proposed BRR medical robot.

## I. Introduction

Bleeding is an inevitable phenomenon during all kinds of surgeries. Clinically, excess blood prevents eyes from good visualization of surgical field and identification of tissue status, and the use of electrocautery enables all unnecessary small volume bleeding to be stopped [1]. Taking neurosurgery [2] as an example, the dynamic blood should be removed and the bleeding spot should be found and then to be coagulated. The task of blood removal contributes a large part of workload of a surgeon. Provided the task of blood removal is fulfilled by a medical robot, the surgeon could concentrate on the more important parts of the current surgery, such as tissue manipulation of cutting and suture *et al.* Therefore, there is need for the autonomy of the task of blood removal.

Task autonomy [3], [4] is a long-term goal of medical robot, and multiple task autonomy together contribute the highest-level medical robot, i.e., the full autonomous medical robot. Currently, the study on task autonomy concentrates on the autonomous completion of a surgical task in one of the surgical stages, like the autonomous tissue suturing in the tissue closing stage after the tumor resection stage [5], intestine anastomosis [6], blunt dissection [7], and tissue retraction [8]. Blood removal operation, resulting from a tremendous amount of the rupture of capillary vessels, is a typical representative of these surgical actions. However, to the best knowledge of the authors, the surgical task of the blood removal by a robot is not addressed to date in existing studies.

Since the blood contour detection (BCD) is the basis of the task of blood removal, it is hardly possible to realize a blood removal robotic system without the result on BCD. In our previous study, for detection bleeding contour on ex vivo porcine skin and during neurosurgical craniotomy, we utilized Mask R-CNN method to find the contour of simulating ex vivo porcine skin and three typical neurosurgical scenarios, including the scalp incision bleeding, skull spot bleeding and dura matter incision bleeding. However, except for our previous work, there is no result on the blood contour detection using a white light camera [9], though there are many studies on intracranial hemorrhage (ICH) on computed tomography imaging modality [10], [11] and bleeding detection in the gastrointestinal tracts in white light imaging modality [12], [13]. Our previous study employs the Mask R-CNN framework [14] to detect the blood contour for a simulating bleeding scenario of the ex vivo porcine skin and three typical neurosurgical scenarios during craniotomy. The obtained results present one of the indispensable prerequisites for the BRR robot.

Kinds of medical robots are designed to verify the feasibility of autonomy of surgical operations. For the autonomy of intestine anastomosis surgery, its supervised autonomy is verified in multiple scenarios, including an ex vivo linear suturing of a longitudinal cut along a length of suspended intestine, an ex vivo end-to-end anastomosis, and an in vivo end-to-end anastomosis of porcine small intestine [6]. For the autonomy of blunt dissection, a study is proposed based on ontology-based surgical sub-task automation to establish an automatic procedure [7]. For the autonomy of tissue retraction in minimally invasive surgery, a novel framework is proposed for planning and executing semi-autonomous [8]. For the autonomy of laparoscopic electro-surgery, a semi-autonomous robotic solution is developed via a 3D endoscope [15]. For the autonomy of soft tissue cutting, an automatic robotic device is designed [16] and the cutting force mechanism is investigated [17]. For manipulation autonomy of a surgical tool, e.g., suturing needle, an automated pick-up method of suturing needles is designed for robotic surgical assistance [18]. For the autonomy of multilateral tumor resection (MTR) surgery, an interchangeable surgical instrument system is designed for the supervised automation of MTR [19]. However, to date, there doesn’t exist any study on either the supervised autonomy or the task autonomy of surgical blood removal, although bleeding is pervasive in almost all kinds of open and minimally invasive surgeries.

In this paper, we will design a robotic system which will fulfill the task autonomy of blood removal surgical action. The robotic system method uses the dual cameras to guide the distal end of the aspirator to follow a planned path determined by the blood contour that is detected by Mask R-CNN framework to suck the blood running out surgical incisions off the tissue surface.

## II. Methods and Procedures

### A. System Structure

**FIGURE 1.**
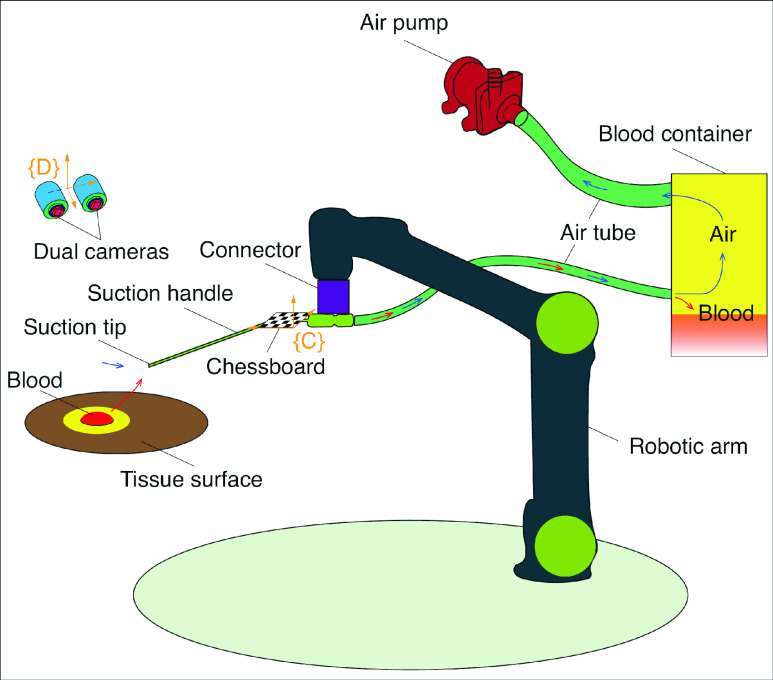
The schematic diagram of the BRR robot for removing blood on tissue surface.

The blood removal robot consists of the following components, i.e., a pair of cameras, a robotic arm, an air pump, a blood container, a mechanical connector, several segments of air tubes, and a suction handle. The schematic diagram of the proposed medical robot system is presented in Fig. 1, where exist a piece of soft tissue and an area of flowed-out blood on its surface, too. The mechanical connector links the suction handle and the robotic arm, and a chessboard is secured to the connector for pinpointing the position of the suction tip of the suction handle. The red and blue arrows in Fig. 1 represent the direction of the fluid air and the fluid blood, respectively. The initial and the final locations of the blood are the tissue surface and the blood container. Let $\{C\}$ and $\{D\}$ denote the coordinate frames of the chessboard and the dual camera, see Fig. 1.

### B. Algorithm Pipeline

**FIGURE 2.**
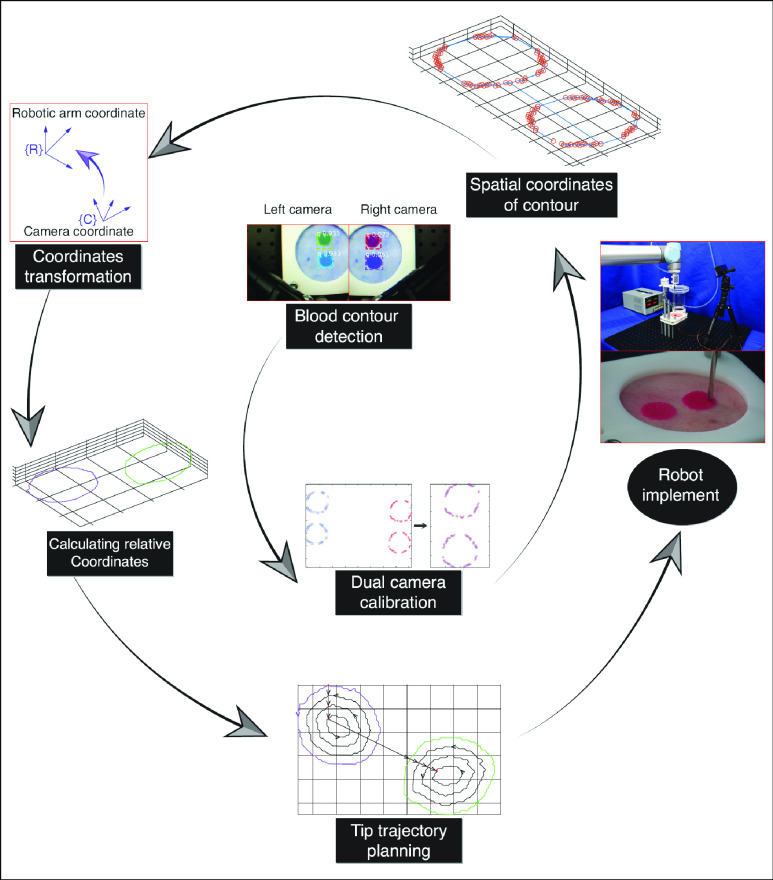
Pipeline of blood removal by the BRR robot.

Using the systematic setup in Fig. 1, the procedure for the blood removal is shown in Fig. 2. First, the Mask R-CNN method is used to detect the contours of the blood region for two images obtained by the dual cameras. Then, the spatial contour of the bleeding region is determined by the segmented closed planar contours, and the spatial coordinates of the 3D contour are also obtained. Next, the coordinates of the spatial contour are transformed to the coordinate frame of the robotic arm, and the differences between the coordinates of the aspirator tip and that of the spatial contour are calculated. For simplification and due to the small dimensions of the blood area, the assumption is adopted that all the coordinates of the spatial contour belong to one common plane. In brief, the detected bleeding contour is treated as a planar curve. According to the assumption, the path planning for the closed contour is implemented, and a planned trajectory for the aspirator tip to follow is obtained. Finally, the robotic arm moves the aspirator tip along the planned path. Simultaneously, the vacuum pump generates the low-pressure zone around the tip and the blood is sucked off the tissue surface from the tip to the blood container.

### C. Blood Contour Detection Via Mask R-CNN

We briefly present the method for blood contour detection, and its full details can be found in our previous work [9]. The Mask R-CNN frame [14] is employed to give the blood contour detection. First, we prepare the image dataset by two means, i.e., simulating bleeding experimental system and video extraction and augmentation. Therefore, we collected a total of 653 raw images of bleeding. Similarly, another 5,000 raw images of bleeding contours are contributed by the simulating bleeding experimental method. Finally, the final data set for the CNN training and verification image date sets consists of 12,600 images following the marking operation on all images.

**FIGURE 3.**
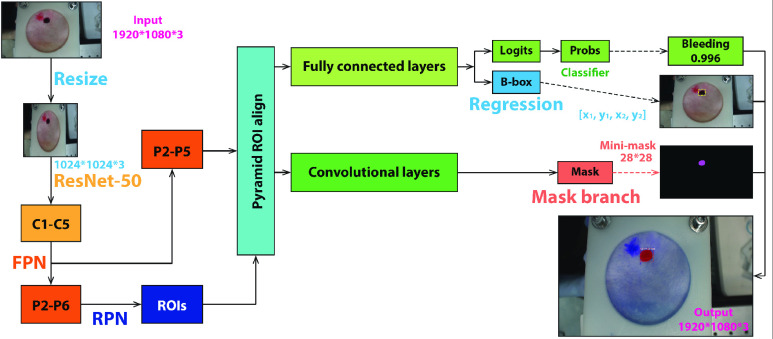
The pipeline of the Mask R-CNN.

The bleeding contour detection adopts the R-CNN mask framework [14]. The initial input images are resized to $1024\times 1024$ pixels of generalized planar dimensions. ResNet-50/101 is then used to extract the feature maps from the input images and the feature extractions are represented by C1-C5. FPN then combines the feature maps and gives rise to the P2-P6 layers of the fused feature. First, the area proposal network (RPN) produces the region of interest (ROI) sub-network, which is fed into the ROI Align layer along with P2-P5, where bilinear interpolation is applied to zoom the image and obtain fixed-dimensional feature maps. The pipeline is then split into two branches, i.e., the mask branch and the classification and bounding box regression branch. The latter branch predicts and generates the bounding box for many classifications. The former branch, a completely convolutional network (FCN), combines the product of the latter in the last step of the process to provide the observed bleeding contour on the output image of the same size, i.e. $1920\times 1080$ pixels, with the original one. A brief pipeline of the Mask R-CNN is shown in Fig. 3.

### D. Spatial Position of Blood Contour

The segmented contours of the left and right images taken by the dual cameras are represented by a pair of point sets. To obtain the spatial location of the detected blood contour from the pair of point sets, point set registration should be addressed first. The coherent point drift (CPD) [20], [21], among various methods, is a widely accepted algorithm for tackling point set registration problems. And the updated algorithm [22], which is derived from the Bayesian formulation setting, proves that the CPD method is of convergence in matching point pairs from the dual point sets. Thus, in this paper, the CPD method is exploited to determine the 3D coordinates of the detected blood contour.

**FIGURE 4.**
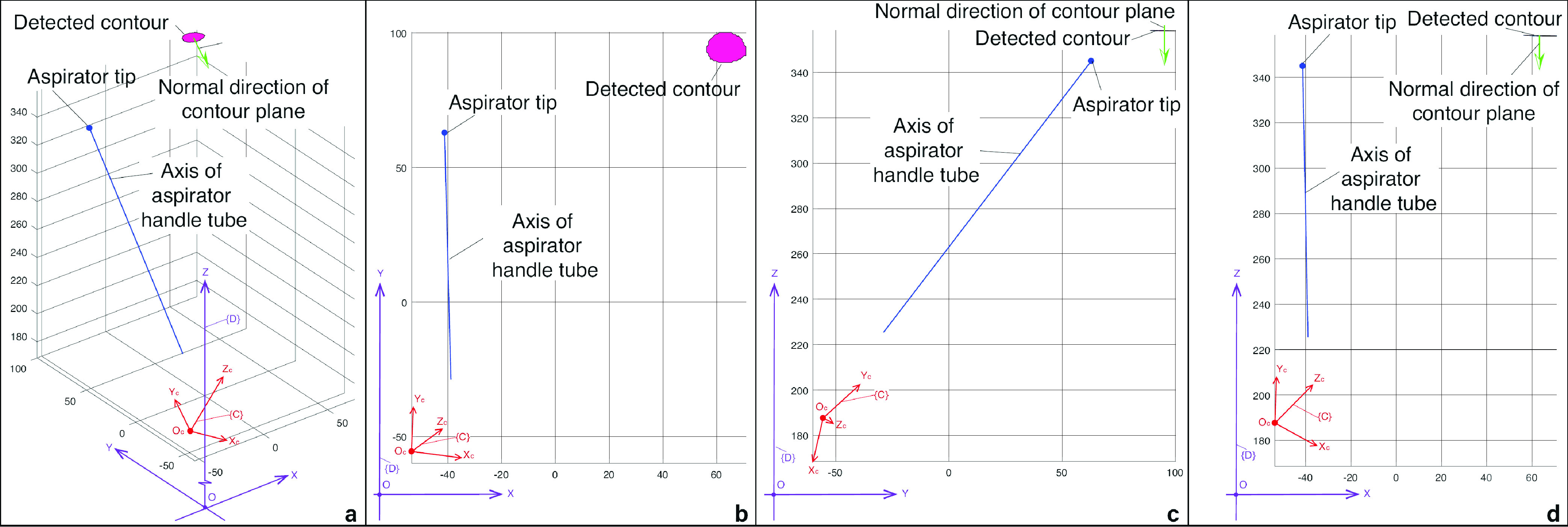
Coordinates frame settings and spatial coordinates of detected blood contour. a. Isometric view. b. X-Y projection view. c. Y-Z projection view. d. X-Z projection view.

After the coordinates of the contour are obtained by CPD, a demonstrative reconstructed 3D contour of the blood region is represented by the red closed spatial curve shown in the top right of Fig. 4. The blue spot represents the aspirator tip, and the blue straight-line denotes the axis of the aspirator handle tube. Using the coordinates of the point set of detected contour, we calculate the average normal direction of the plane which contains the blood contour. The green arrow represents the average normal direction of the plane of the detected contour. The normal direction guarantees that the aspirator’s cylinder is vertical to the blood area and the nearest point between the robot and the soft tissue is the suction tip. A path planning algorithm, in the following section, gives a planned trajectory inside the detected contour for the tip to track. During the tip moves along the planned trajectory, the aspirator axis keeps the same direction as the normal direction of the contour plane.

### E. Trajectory Planning Method

After the blood region is obtained in the former steps, the blood removal trajectory is planned in this part. There are various methods to generate a trajectory which covers a closed area for diverse purposes, such as the treatment planning of laser ablation of tissue [23], the path planning of additive manufacturing [24], and an online trajectory planning method for autonomous surgery [25]. While for the bleeding scenario, often the bleeding spot exists near the center of the contour, and seldom the bleeding incision is located on the edge of the blood contour. Provided the aspirator tip starts from the center of the contour, the running-out blood near the incision will be removed firstly. However, after all the planned trajectory is traced, more blood will flow out from the incision near the center of the contour and cover some regions inside the contour once again. On the contrary, when the path is traced from its edge to the center, the aspirator tip will finally approach the bleeding incision, avoiding the disadvantage of the former manner. Thus, the design of the trajectory planning algorithm adopts the latter principle.

**FIGURE 5.**
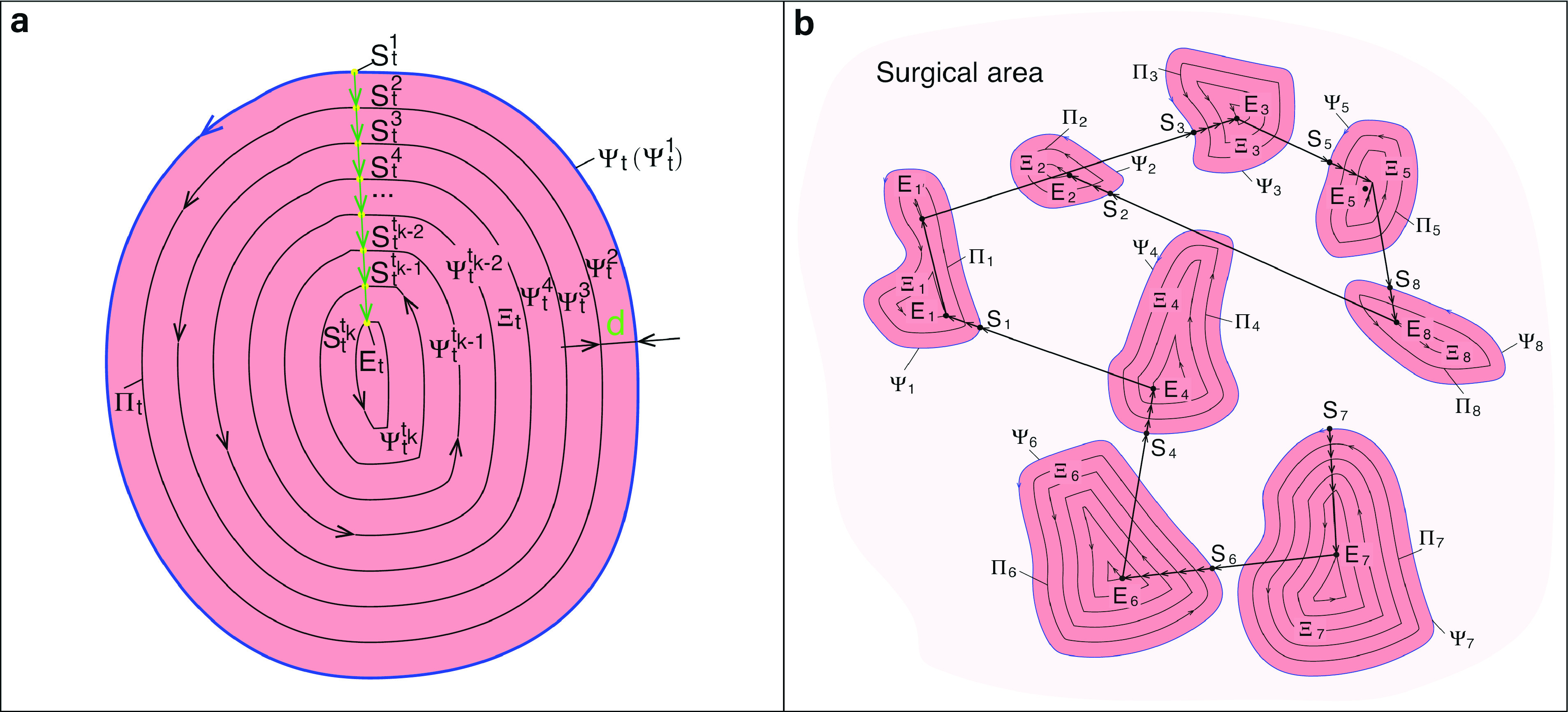
**Trajectory planning algorithm**. a. Lower-level path planning algorithm. b. Higher-level path planning algorithm.

According to the aforementioned assumption of the contour being a planar closed curve, the task of the path planning for the blood contour detection is treated as a planar path planning problem. The schematic of the trajectory planning method is shown in Fig. 5. Denote the detected contours by $\Psi _{t}$, $1\leq t \leq T$, $T \in \mathbb {N}$, and $T$ is the amount of the detected contours in surgical region. Let $\Xi _{t}$ denote the enclosed area by the contour $\Psi _{t}$. Denote the start point of the planned path $\Psi _{t}$ by $S_{t}$, which is also the end point of $\Psi _{t}$, and denote the planned trajectory by $\Psi ^{i_{t}}_{t}$, $1\leq i_{t} \leq k_{t}$, $i_{t} \in \mathbb {N}$, which is a group of inner equal distance contours. Denote the distance between $\Psi ^{i_{t}}_{t}$ and $\Psi ^{i_{t}+1}_{t}$ by d which is determined by the low-pressure at the aspirator tip. In this study, considering the positional error between the tip and the true contour of the blood region, $d$ is equal to 2mm which is 1mm smaller than the diameter 3mm of the aspirator tube. See Section III-D for more details about error analysis.

Denote the area of the regions by $A_{t}$, $t \in T$. The cleaning order of the multiple blood areas are determined by the area of the regions. The bigger the area, the higher the priority of the blood cleaning of the region. Providing the areas of several regions share a same value, the sorting priority is determined by the left-to-right order of the columns. For example, in Fig. 5b, we present the detected blood contour $\Psi _{1}$ and its inner equal-distance parallel curves $\Psi _{i}$, $i\in [{2,8}]$, $i \in \mathbb {N}$. And the removal order of the multiple blood regions is $\Xi _{7} \rightarrow \Xi _{6} \rightarrow \Xi _{4} \rightarrow \Xi _{1} \rightarrow \Xi _{3} \rightarrow \Xi _{5} \rightarrow \Xi _{8} \rightarrow \Xi _{2}$ due to the descending order of their areas, i.e., $A_{7} \geq A_{6} \geq A_{4} \geq A_{1} \geq A_{3} \geq A_{5} \geq A_{8} \geq A_{2}$. The procedure for blood removal, i.e., determining the contour $\Psi ^{i_{t}}_{t}$ and the start points $S_{t}$ of $\Psi ^{i_{t}}_{t}$, can be found in **Algorithm 1** presented below.

Algorithm 1 **Path Planning Algorithm for Blood Removal**
1: Find all the blood contours $\Psi _{t}$, (also $\Psi _{t}^{1}$), $1\leq t \leq T$, $T \in \mathbb {N}$,
2: Sort T contours in descending order according to their areas, $A_{i} \geq A_{j}$, $\forall i\geq j, i,j \in T$,
3: Set the distance $d$,
4: Find the group of inner equal distance contours $\Psi ^{i_{t}}_{t}$, $2\leq i_{t} \leq k_{t}$, $i_{t} \in \mathbb {N}$, inside $\Psi _{t}$ with the distance $d$,
5: **if** $t$=1 **then**
6: Find the start point $S_{t}^{1}=(x_{j},y_{j})\in \Psi ^{1}_{t}$, $x_{j}\geq x_{s}$, $\forall (x_{s},y_{s})\in \Psi ^{1}_{t}$,
7: Find the nearest point $S^{k}_{1}$ to $S_{1}^{1}$, $S^{k}_{1} \in \Psi ^{k}_{1}$,
8: Connect $S_{1}^{1}$ and $S^{k}_{1}$, and obtain line $\overline {S_{1}^{1}~S^{k}_{1}}$,
9: Determine the intersection points $S^{i_{t}}_{1} \in \Psi ^{i_{t}}_{1}$, $S^{i_{t}}_{1} \in \overline {S_{1}^{1}~S^{k}_{1}}$,
10: **else**
11: Find the nearest point $S^{k_{t}}_{t} \in \Psi ^{k_{t}}_{t}$ to the point $S^{k_{t-1}}_{t-1} \in \Psi ^{k_{t-1}}_{t-1}$, $1\leq t \leq T$, $t \in \mathbb {N}$,
12: Connect $S^{k_{t}}_{t} $ and $S^{k_{t-1}}_{t-1}$, and obtain line $\overline {S^{k_{t}}_{t} S^{k_{t-1}}_{t-1}}$,
13: Determine the intersection points $S^{i_{t}}_{t} \in \Psi ^{i_{t}}_{t}$, $S^{i_{t}}_{t} \in \overline {S^{k_{t}}_{t} S^{k_{t-1}}_{t-1}}$,
14: **end if**
15: **for** $j_{t} =1\rightarrow k_{t}$ **do**
16: Move the aspirator tip along the contour $\Psi ^{j_{t}}_{t}$ from $S^{j_{t}}_{t}$ and return to $S^{j_{t}}_{t}$,
17: **if** $j_{t} =k_{t}$ **then**
18: Move the aspirator tip from $S^{j_{t}}_{t} \in \Psi ^{j_{t}}_{t}$ to $S^{1}_{t+1} \in \Psi ^{1}_{t+1}$,
19: **else**
20: Move the aspirator tip from $S^{j_{t}}_{t} \in \Psi ^{j_{t}}_{t}$ to $S^{j_{t}+1}_{t} \in \Psi ^{j_{t}+1}_{t}$,
21: **end if**
22: **end for**

The distance between the suction tip of the aspirator should be short enough so that the air in the low-pressure zone around the tip will be dragged into the suction tip. For the sake of safety, the distance between the tip and the curve is 1mm, which is experimental verified effective for suction of the blood. Also, the axis of the long cylindrical suction handle is normal to the tissue surface.

### F. Simulating Bleeding Experimental System

**FIGURE 6.**
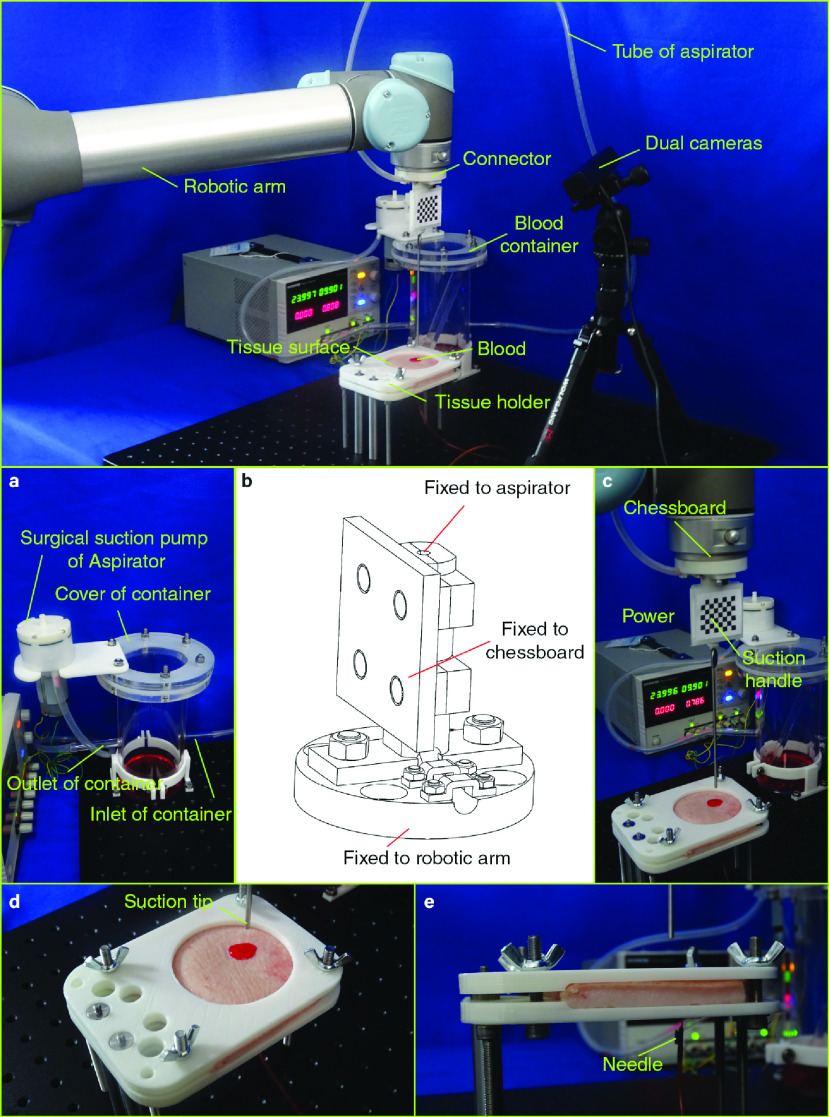
**The BRR prototype**. a. Simulating aspirator system. b. Connector of BRR. c. Frontal end of aspirator of BRR, and the experimental setup of the simulating single bleeding area on a piece of ex vivo porcine skin. d. Ex vivo porcine tissue holder. e. Needle inserted to simulate bleeding.

A simulating bleeding experimental system is built to verify the feasibility of the blood removal of the proposed robot, and to obtain the images as the training data of the Mask R-CNN. We first present the bleeding contour detection results of a simulating single point bleeding on a piece of ex vivo porcine skin. The simulating bleeding experimental system is presented in Fig. 6, and Fig. 6e demonstrates the top and bottom tissue holder frames which are 3D printed mechanical structures for fixing the porcine skin and its underlayer tissue. The observable porcine tissue area with a diameter of 10 cm in Fig. 6d, which is similar to that of the exposed surgical region of neurosurgical craniotomy.

The flow rate of the pump is 13 L/min, and the ultimate air pressure of the pump is −50KPa. The length and diameter of the suction cylinder are 15cm and 3 mm, respectively. The volume of the blood collector is 4.5 Liters, and the thickness of its wall is 10 mm, which is strong enough against its low pressure. The dimensions of the pump are $\phi 60$ mm$\times 112$ mm.

## III. Results

With the proposed robotic system, we carried out a series of ex vivo animal tissue bleeding experiments on pieces of porcine skin. The main algorithm steps and consecutive procedures of blood removal for a demonstrative procedure of the multiple blood areas by the robot are presented. The flowed-out blood is removed thoroughly through the aspirator fixed to the end of the robotic arm.

### A. Prototype Built

With the preparations on the mechanical structures and the algorithms, it is ready to develop the prototypical blood removal robot. The built robot is shown in Fig. 6. The components of the built prototype are corresponding to the counterparts in the schematic diagram of the robot in Fig. 1. The simulating aspirator system, shown in Fig. 6a, contains a suction pump, a container cover, an outlet and an inlet. The detailed design of the connector is shown in Fig. 6b, and the dimension of the connector is $\phi 64$ mm$\times 73$ mm. The frontal end of the aspirator system is shown in Fig. 6c, where the connector binds the chessboard, the robotic arm and the aspirator together. The needle in Fig. 6e indicates the position of bleeding spot, and the number of the needle is equal to the count of the blood regions. The coordinate of the aspirator tip and the origin $O_{c}$ of the coordinate frame $\{D\}$ are {–41.28 mm, 62.86 mm, 284.99 mm} and {–18.70 mm,–59.97 mm, 108.57 mm}.

### B. Detected Contour

**FIGURE 7.**
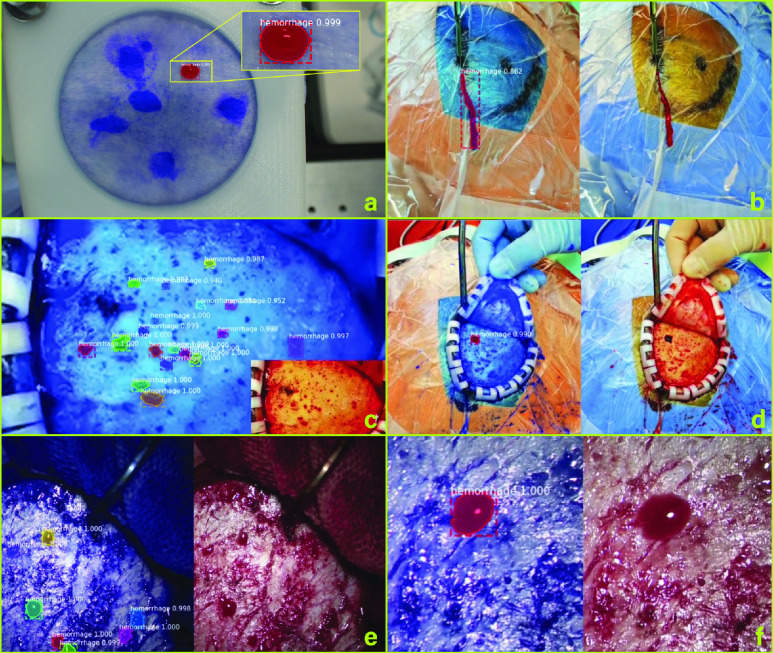
**Blood contours detected.** a. Simulating bleeding scenario on a piece of ex vivo porcine skin. b. Real neurosurgical blood cases. c. Multiple areas of skull bleeding. d. A single area of skull bleeding. e. Multiple areas of dura matter incision. f. Single bleeding area of dura matter incision.

By the aid of the Mask R-CNN framework, we detected the blood contours in both the simulating and real bleeding scenarios, shown in Fig. 7. The former is a single bleeding area on a piece of simulating bleeding ex vivo porcine skin. The latter consists of five typical true neurosurgical scenarios, i.e., a single area of skull bleeding, multiple areas of skull bleeding, multiple areas of dura matter incision, single bleeding area of dura matter incision, and single bleeding area of scalp incision.

### C. A Demonstrative Example

**FIGURE 8.**
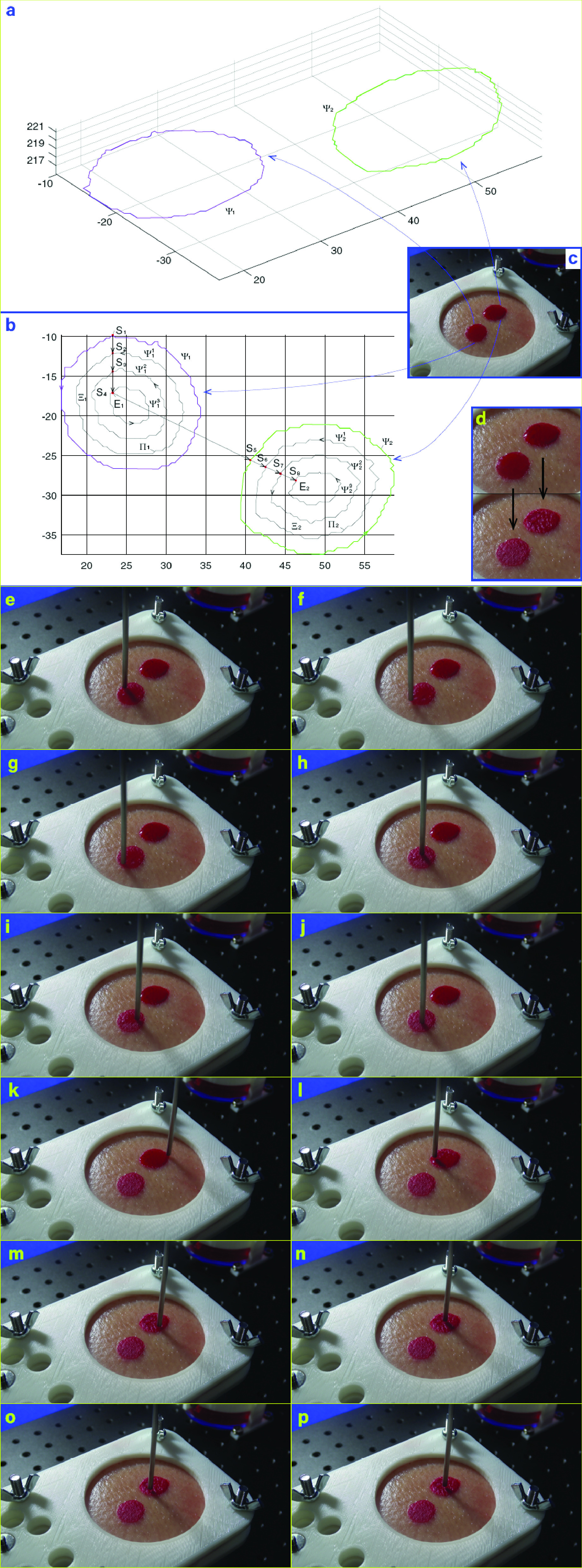
A demonstrative autonomous blood removal experiment by the proposed robot. a. Detected contour coordinates. b. Planned path. c. Blood contours before removal. d. Comparison between the original and blood removed statuses. e–p. A series of frames captured from video.

A consecutive procedure of blood removal by the proposed robot is presented in Fig. 8. The spatial coordinate of the blood contour and the reconstructed 3D contour of the detected blood region are given in Fig. 8a, and taking two blood regions as example, the path planned for aspirator tip to follow is provided in Fig. 8b. The real scenario of the demonstrative multiple blood regions is imaged and shown in Fig. 8c.

The demonstrative blood removal procedure by the robot for multiple blood regions is shown in Fig. 8e to Fig. 8p. From Fig. 8d, one can identify the initial state before the blood removal operation and the final scenario after removal of the blood areas. One can find that most of the blood is immediately sucked off when the tip reaches the contour at the initial position for both regions. Finally, the blood area is cleaned and no blood is left. The processing time for the experiment in Fig. 8 is 18.9 seconds, which is not the minimal value since the robot arm did not run at its highest velocity.

The removal order of the multiple blood areas for the scenario in Fig. 8b is $\Psi _{1} \rightarrow \Psi _{2} $. First, the aspirator tip is moved by the robotic arm to follow the curve $\Psi _{1}$ and its inner equal-distance parallel curves $\Psi _{1}^{1}$, $\Psi _{1}^{2}$, $\Psi _{1}^{3}$ from the start point $S_{1}$ to the end point $E_{1}$. Next, the tip moves from $E_{1}$ to the start point $S_{2}$ of the second contour $\Psi _{2}$. Then, the aspirator tip follows the curve $\Psi _{2}$ and its inner equal-distance parallel curves $\Psi _{2}^{1}$, $\Psi _{2}^{2}$, $\Psi _{2}^{3}$ from the start point $S_{2}$ to the end point $E_{2}$. For each contour of $\Psi _{i}^{j}$, $i=1,2,j=1,2,3$, the start point is also the end point.

### D. Precision Analysis

**FIGURE 9.**
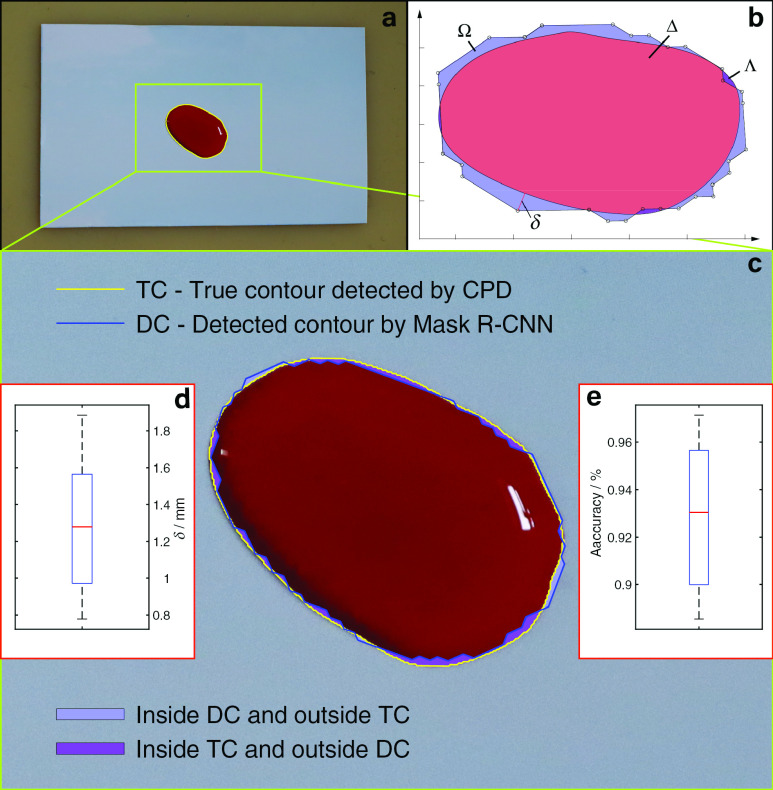
Analysis of contour detection accuracy. a. Original blood contour. b. Coordinates of the detected and true contours. c. Error between the planned path and the true contour of the blood region.

For precision analysis of the contour detected by the proposed method, a top view of the flowed-out blood, shown in Fig. 9a, is obtained for identifying its true contour, which is proved by several surgeons and can be taken as the gold standard of the precise edge of the blood region.

The calculation of the detection accuracy of the blood contour by Mask R-CNN is shown in Fig. 9b. The accuracy of blood contour detection is defined by

\begin{equation*} P=\dfrac {(S_{\Omega }+S_{\Lambda })}{(S_{\Lambda }+S_{\Delta })}\times 100=\dfrac {(S_{\Omega }+S_{\Lambda })}{S_{\Xi }}\times 100\%\tag{1}\end{equation*}

where $S_\Xi =S_\Lambda +S_\Delta $ is the area of detected contour by Mask R-CNN. $S_{\Omega }$ is the area belonging to the detected contour (DC) and not contained by the true contour (TC). $S_{\Lambda }$ is the area inside TC and is not contained by DC. $S_{\Delta }$ is an intersection area enclosed by both TC and DC. The median of the detected intersection of the contour is 93.03% in Fig. 9e.

Besides, there exists a nontrivial scenario, where the distance between the tip and the true contour is overlarge and the area is comparatively small. To avoid the possibility of missing removal of blood area in this case, another index for evaluating the accuracy of the detected contour is provided, i.e., the maximum of Hausdorff distance [26] between TC and DC denoted by $\delta $ in Fig. 9b. The median of the maximal distance between TC and DC is 1.27 mm in Fig. 9d.

The positional error between the planned trajectory and the aspirator tip is determined by the position accuracy of the robotic arm, and the error is not larger than 0.1 mm which is guaranteed by the manufacturer Universal Robot® and also verified in our laboratory. Thus, the median and the maximal compound positional errors between the tip and the true contour are 1.37 mm and 1.99 mm, which is equal to the sum of 0.1 mm and the median 1.27 mm and 1.89 mm, respectively. Despite these positional errors, the blood regions in all the experiments were removed by the robot, due to the existence of the spatial low-pressure zone enclosing the aspirator tip.

## IV. Discussions

We present, for the first time to the author’s knowledge, the schematic diagram of the blood removal robotic system and its prototype. The feasibility of the system design in blood removal is confirmed by the simulating bleeding experiments on the ex vivo porcine skin. The blood contour is detected and the spatial coordinates of the contour are reconstructed. Then, the path of the aspirator tip is planned and implemented by the robotic arm. Finally, the blood is removed thoroughly by the proposed robot. Accordingly, the robotic system is ready to be integrated into a high-autonomy medical robot which needs the capability of blood removal.

The blood removal trajectory by surgeon depends on their experiences and the overall principle for blood removal is that the dynamic bleeding should be treated and static bleeding area is free of operation. Besides the presented scanning path planning algorithm using the group of equal distance parallel curves, there are kinds of algorithms which give inspiration to design a planned trajectory for the aspirator tip to follow. For example, there are various path planning methods that individually generate a scanning path for a closed area, e.g., the raster path scanning method [27, 28], the zigzag path generating method [29, 30], the contour path [31], continuous path [32], spiral path [24] and hybrid path [33]. However, due to the different fields these results belong to, removal of irrelative factors such as flying aerodynamics [34] and addition of reasonable factors such as the bio-fluid mechanics [35] should be taken into consideration. At the same time, the comparison between the accuracy of the path planning methods will give clue on selecting an appropriate method.

For safety consideration, there is no contact between the brain tissue and the tip of the aspirator, and the distance between the tip and the curve is set to be 1 mm, which is experimentally confirmed to be sufficient for a blood cleaning robot. The distance could be under further investigation and confirmed, provided the theoretical fluid mechanism model between the spatial form of the low-pressure zone and the fluid viscosity of the blood is derived based on the fluid mechanism theorems [35]. However, the distance between the aspirator tip and blood contour might be a bigger value for there is a low-pressure area near the aspirator tip due to the vacuum pump.

An appropriate parameter $d$ in Fig. 4a is key for determining whether the blood within the detected contour could be removed thoroughly or not. Likewise, the distance ${d}$ affects the operational time of the blood removal task. For example, a decreased value of ${d}$ will increase the length of the curve and also give rise to the processing time. Simultaneously, more time means more blood running out of the incision. One of the possible solutions is that the distance $d$ between the tip and the blood surface was relative to and depending on the overall contour size. It is worthy of more systematic investigations in future study. Also, similar to the path planning [34], the optimization algorithm for finding the optimal distance ${d}$ should be addressed for a better performance of the robot.

Reduction of surgical time is beneficial for both surgeons and patients. However, the processing time is not one of the key indexes for a feasibility study of an autonomous medical system, including the automated blood removal robotic system in this study. For example, the task time is not mentioned by the studies on the autonomy of medical robots [6, 8, 15-19, 25]. In fact, the processing time could be minimized by accelerating the motion speed of the tip to the maximal velocity of the robot arm. The time cost of the blood removal procedure consists of three parts. The first part is the time cost attributed to the robot executing its planned movement, the second part is the processing time for the detection of blood contours, and the third part is the time attributed to breaking them down to inner sub-contours. For the first part, the time cost could be reduced by using a light-weight robotic arm or designing and building a customized robotic arm, or by optimizing the algorithmic implementation through the utilizing the hardware-level instructions. For the second part, the time cost could be reduced by optimization of the contour detection algorithm or boosting the algorithm implement on a hardware, like GPU or FPGA. For the last part, the time cost could be reduced by reducing the amount of the sub-contours through investigating the interaction mechanism between the air pressure at the tip and the blood fluid.

Due to high occurrence of bleeding during surgeries, often the task of the blood removal and other surgical operations are alternately implemented, like the coagulation of the bleeding incision with a bipolar coagulation electrosurgical unit in neurosurgery. The blood removal system in the study could be integrated with a bipolar coagulation electrosurgical unit like the design of the combination suction-cautery tip designed for neurological surgery [36]. The integration will release surgeon, to a certain extent, from heavy workload, and allow surgeons to allocate more time to consider sophisticated tasks, like tumor resection.

The planned path is treated as a planar one, though not all points of the blood contour are precisely in one common plane. The assumption that the blood contour is planar is reasonable for small bleeding area. And this assumption would be with some error when the bleeding area is large on a curved surface. The assumption could be released by more investigations on the shape of the surgical area. In fact, due to the low-pressure zone near the aspirator tip, this assumption is reasonable and all the blood within the contour was removed actually like the results in Fig. 8d to Fig. 8p. Furthermore, provided to escape from the planar-path assumption, one can install a distance sensor to the tip of the aspirator, and then a feedback controller with real-time measurement of the distance between the tip and points of the contour could be used to keep a desired distance between the tip and the surface. Besides, it is better to develop an auxiliary method to detect that all blood has been removed, and thus stop the robot from carrying out the rest of its planned trajectory.

In many proof of principle studies on the surgical autonomy, various phantoms, not living animal or human patient are employed, such as porcine small intestine [6], porcine skin [17], and in vitro tissue-mimicking models [37]. Likewise, in the current study, we use the simulation bleeding scenario on the pieces of ex vivo porcine tissue to demonstrate the feasibility of the task autonomy of the blood removal operation. Also, due to the differences between the textures of various sorts of soft and hard tissue during surgeries, the precision of the blood contour detection might fluctuate. Therefore, the applicability and precision of the blood contour detection method will be checked and optimized for multiple types of surgical bleeding scenarios. The comparison between the proposed method and the standard process [38] of blood removal and coagulation should be investigated after the proposed autonomous blood removal method is combined with an autonomous coagulation method in an autonomous blood removal and coagulation robotic system.

## V. Conclusion

A blood removal medical robot is designed and built, for the first time as far as we know, for the surgeries involving tissue bleeding. Through the demonstration of a group of ex vivo porcine skin experiments, it is proved that the proposed blood removal robot can accomplish the blood cleaning task. Therefore, the resultant system lays one of the foundations for a higher-level surgical task. For example, the robot could be integrated into a medical robotic system, which fulfills a surgical task relying on blood removal operation in multiple surgical stages. In the following next study, we are redesigning the mechanical structures of the robot aiming to integrate the robot with multiple functions, including the coagulation task of bleeding incision by a bipolar electrical unit.
